# Nanotechnology and surgical neurology

**DOI:** 10.4103/2152-7806.69384

**Published:** 2010-09-16

**Authors:** Rajiv Saini, Santosh Saini

**Affiliations:** Department of Periodontology & Oral Implantology, Rural Dental College-Loni, Maharashtra, India; 1 Department of Microbiology, Rural Dental College-Loni, Maharashtra, India

Sir,

Nanotechnology can be defined as the science and engineering involved in the design, synthesis, characterization, and application of materials and devices whose smallest functional organization, in at least one dimension, is on the nanometer scale or one billionth of a meter, and the promise that nanotechnology brings is multifaceted, not only offering improvements to the current techniques but also providing entirely new tools and capabilities.[3] Nanotechnology as a science has evolved from notions and speculation to emerge as a prominent combination of science and engineering, which stands to impact innumerable aspects of technology.[2] Various nanotechniques will make substantial contributions to the advancement of neurosurgery in the near future, such as (1) nanomanipulation, (2) nanoimaging, (3) non-surgical nanorepair, and (4) nanoneuromodulation. Nanomanipulation includes several techniques for performing what might be called “surgery” on the nervous system at the level of the neuron, neuronal processes, or intracellularly. Nanoimaging refers to viewing the nervous system (and its disorders) at the cellular or subcellular level. Non-surgical nanorepair considers substances (usually manufactured rather than naturally occurring) with impressive properties to promote axonal regeneration, halt deleterious processes (e.g., hemorrhaging), and extend neuronal (and organ to organism level) lifespan. Nanoneuromodulation refers to interacting with the nervous system at the nano/neuronal level – either monitoring or stimulating – in order to affect the nervous system’s electrical and/or electrochemical (e.g., neurotransmitter) function.[1] Nanoimaging image guidance is used as an aid in the surgical localization of pathology, minimization of incisions, and improvement of surgical intervention outcomes. Molecular imaging modalities, based on quantum dots and magnetic nanoparticle technology, have been extended to the imaging of intracranial neoplasms. Neuromodulation therapies directly address the root of the problem in patients with spina bifida and a neurogenic bladder, namely, the abnormal relationship between the nerves and the bladder wall. These therapies include transurethral bladder electrostimulation, sacral neuromodulation, and neurosurgical techniques such as selective sacral rhizotomy and artificial somatic–autonomic reflex pathway construction. Nanomanipulation can be applied to the scientific exploration of mesoscopic phenomena and the construction of prototype nanodevices. One of the chief challenges that the neurosurgeons face is that due to the complicated and delicate structure of the brain, it is enormously hard and risk-laden to operate on this organ. Nanobiotechnology introduces another dimension in robotics, leading to the development of nanorobots also referred to as nanobots. Instead of performing procedures from outside the body, nanobots will be miniaturized for introduction into the body through the vascular system or at the end of catheters into various vessels and other cavities in the human body. Surgical nanobot, programmed by a human surgeon, could act as an autonomous on-site surgeon inside the human body. Various functions such as searching for pathology, diagnosis and removal or correction of the lesion by nanomanipulation can be performed and coordinated by an on-board computer. Such concepts, once science fiction, are now considered to be within the realm of possibility. Nanorobots will have the capability to perform precise and refined intracellular surgery which is beyond the capability of manipulations by the human hand. Surgical “nanobots” outfitted with operating instruments would have the motility and thoroughness that is not available to direct manipulations by a human hand, and operations using nanobots could be less invasive than the current methods thus helping with faster recovery. Nanotechnology and nanoengineering stand to produce significant scientific and technological advances in diverse fields including medicine and physiology. For applications in medicine and physiology, these materials and devices can be designed to interact with cells and tissues at a molecular (i.e., subcellular) level with a high degree of functional specificity, thus allowing a degree of integration between technology and biological systems, not previously attainable. It should be appreciated that nanotechnology is not in itself a single emerging scientific discipline but rather a meeting of traditional sciences such as chemistry, physics, materials science, and biology to bring together the required collective expertise needed to develop these novel technologies. True to the highly interdisciplinary nature of this area of research, it is important that technological advancements occur in conjunction with basic and clinical neuroscience advances. Therefore, three things that must occur in parallel for nanotechnology applications in neurology and neurosurgery to come to fruition are:[4]

1. advances in chemistry and materials science that produce ever more sophisticated synthetic and characterization approaches
2. advances in the molecular biology, neurophysiology, and neuropathology of the nervous system and
3. the design and integration of specific nanoengineered applications to the nervous system, which take advantage of the first two points.

As use of nanotechnology infuses all forms of technical and medical research, clinical applications will carry on to emerge. Surgeon and clinical physician of the present and future must take an active function in shaping the plan and research of nanotechnologies to ensure maximal clinical relevance and patient benefit. Eventually, the goal is to expand technologies that directly or indirectly support in providing a liberal setting to the advancement of neurosurgery in the near future. Applications of nanotechnology in neuroscience are already having significant effects, which will continue in the foreseeable future. Short-term progress has benefited *in vitro* and *ex vivio* studies of neural cells, often supporting or augmenting standard technologies. These advances contribute to both our basic understanding of cellular neurobiology and neurophysiology, and to our understanding and interpretation of neuropathology. Although the development of nanotechnologies designed to interact with the nervous system *in vivo* is slow and challenging, they will have significant, direct clinical implications.
